# Sustaining essential health services in Lao PDR in the context of donor transition and COVID-19

**DOI:** 10.1093/heapol/czad090

**Published:** 2024-01-23

**Authors:** Eunkyoung Kim, Yu Lee Park, Ying-Ru Lo, Bounserth Keoprasith, Suphab Panyakeo

**Affiliations:** Health System Development team, World Health Organization Country Office for the Lao People’s Democratic Republic, 125 Saphanthong Road, Unit 5, Ban Saphanthongtai, Sisattanak District, Vientiane Capital 0103, Lao People’s Democratic Republic; Health System Development team, World Health Organization Country Office for the Lao People’s Democratic Republic, 125 Saphanthong Road, Unit 5, Ban Saphanthongtai, Sisattanak District, Vientiane Capital 0103, Lao People’s Democratic Republic; WHO Representative to Lao People’s Democratic Republic, World Health Organization Country Office for the Lao People’s Democratic Republic, 125 Saphanthong Road, Unit 5, Ban Saphanthongtai, Sisattanak District, Vientiane Capital 0103, Lao People’s Democratic Republic; Department of Planning and Finance, Ministry of Health, Ban Thatkhao, Sisattanack District, Rue Simeuang, Vientiane Capital 0103, Lao People’s Democratic Republic; Department of Planning and Finance, Ministry of Health, Ban Thatkhao, Sisattanack District, Rue Simeuang, Vientiane Capital 0103, Lao People’s Democratic Republic

**Keywords:** Health financing, health policy, health sector reform, health systems, primary health care, Lao PDR

## Abstract

Lao People’s Democratic Republic (Lao PDR) aims at graduating from least developed country status by 2026 and must increase the level of domestic financing for health. This paper examines how the government has prepared for the decline of external assistance and how donors have applied their transition approaches. Adapting a World Health Organization (WHO) framework, reflections and lessons were generated based on literature review, informal and formal consultations and focus group discussions with the Lao PDR government and development partners including budget impact discussion. The government has taken three approaches to transition from external to domestic funding: mobilizing domestic resources, increasing efficiency across programs and prioritization with a focus on strengthening primary health care (PHC). The government has increased gradually domestic government health expenditures as a share of the government expenditure from 2.6% in 2013 to 4.9% in 2019. The Ministry of Health has made efforts to design and roll out integrated service delivery of maternal, newborn, child, and adolescent health services, immunization and nutrition; integrated 13 information systems of key health programs into one single District Health Information Software 2; and prioritized PHC, which has led to shifting donors towards supporting PHC. Donors have revisited their aid policies designed to improve sustainability and ownership of the government. However, the government faces challenges in improving cross-programmatic efficiency at the operational level and in further increasing the health budget due to the economic crisis aggravated during Coronavirus disease 2019 (COVID-19). Working to implement donor transition strategies under the current economic situation and country challenges, calls into question the criteria used to evaluate transition. This criterion needs to include more appropriate indicators other than gross national income per capita, which does not reflect a country’s readiness and capacity of the health system. There should be a more country-tailored strategy and support for considering the context and system-wide readiness during donor transition.

Key messages

- The government of Lao People’s Democratic Republic has taken three approaches to transition from external to domestic funding for health: mobilizing domestic resources, increasing efficiency across programs and reprioritizing activities with a focus on strengthening primary health care.
- Efforts have been made by the government and development partners to redirect external assistance for health away from supporting vertical programs and more towards health system strengthening by shaping partners’ policy directions to align with government priorities.
- In order to secure the delivery of essential public health programs and improve efficiencies in the health system, system level action is needed with support from development partners by reducing fragmentation through pooling and strategic allocation of funds and service integration.
- The criteria used by donors to evaluate transition are called into question as the country works to implement donor transition strategies under the current economic situation and country challenges. Additional indicators other than gross national income per capita should be used to reflect a country’s readiness and capacity of the health system to transition from external donor support. Such evaluation can inform a country-tailored strategy for smooth transition and support considering the country context and system-wide readiness during the transition.

## Introduction

Donor transition can have significant implications affecting health systems from a health financing perspective (Ministry of Health of Lao PDR, 2021a). From the literature, there is a need to better understand the impact of donor transition on country health systems and on the sustainability of essential health services (EHS) (Huffstetler *et al.*, 2022). This paper provides a critical review of the approaches Lao People’s Democratic Republic (Lao PDR) has taken to prepare for donor transition.

Lao PDR is expected to graduate from the least developed country status by 2026, given the rapid economic growth over the past 10 years before the Coronavirus disease 2019 (COVID-19) economic contraction. This has initiated the transition from external financing and has resulted in increased domestic funding. However, external funding was still 22.5% of total health expenditure in 2019 (Ministry of Health of Lao PDR, 2021b), much higher than other neighbouring countries (15.5% in 2019 in World Health Organization (WHO) Western Pacific Region) (World Health Organization, 2022a).

The reduction of external funding in the country, along with the global geopolitical situation, will pose challenges in sustaining EHS, including routine immunizations. For instance, the forecasted economic growth for 2022 decreased to 2.5%, from an earlier projection of 3.8% (World Bank, 2022), and inflation rose from less than 2% to 41.3% from February 2021–2023 (Bank of Lao PDR, 2023). As a result, essential commodity prices for fuel, food and medicines have increased sharply, and the de-facto total government budget allocated for health lost half its purchasing power. In addition, the cost in currency Lao Kip increased 30%, reflecting the fragility and sustainability challenges of fulfilling co-financing requirements that are in US dollars. As such, the 2023 health budget allocation for operational costs will be completely utilized by the national health insurance (NHI) and co-financing, crowding out other program activities (Department of Planning and Cooperation/MOH of Lao PDR, 2023). Non-wage budget cuts have also been made at the central and provincial levels (National Assembly of Lao PDR, 2020), which further threatens sustainability of EHS.

External funding channelled through vertical programs often operates independently from the national health system, which increases inefficiency in resource use and compromises effective management (Kutzin *et al.*, 2018). As Lao PDR transitions away from donor financing, it is now faced with these challenges and needs to explore solutions for better alignment and integration of donor supported areas within the overall health system. Donors commonly use gross national income (GNI) per capita to evaluate donor transition (Kallenberg *et al.*, 2016; McDade *et al.*, 2020); however, the usefulness of this indicator to evaluate a country’s readiness for donor transition is called into question.

**Figure 1.**
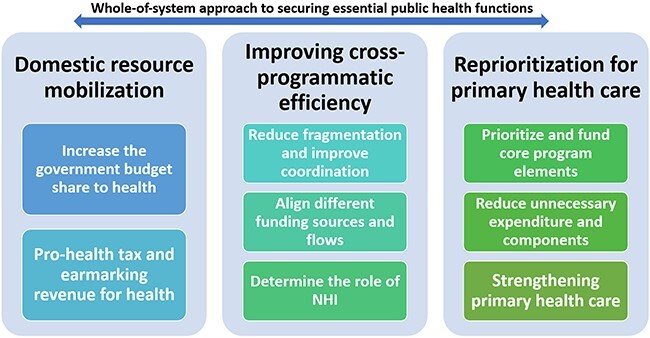
WHO framework to the Lao PDR context based on the 2018 WHO Regional framework for action on transitioning to integrated financing of priority public health services in the Western Pacific

Note: NHI, National Health Insurance.

The objective of this paper is to review the Lao PDR Ministry of Health (MOH)’s donor transition strategy and donors’ approaches to transition. Literature reviews of peer-reviewed articles, grey literature, meeting reports and government documents including the National Health Accounts report for 2019 were conducted. Lessons were generated based on informal and formal consultations and focus group discussions with the government and donors. Using the 2018 Regional framework on transitioning to integrated financing of priority public health services in the Western Pacific (World Health Organization, 2018), three transition strategies taken by Lao PDR are discussed below (Figure 1).

## Implementation

Based on the Health Financing Strategy 2021–2025 (Ministry of Health of Lao PDR, 2021a) and the Health Sector Reform Strategy 2021–2030 (Ministry of Health of Lao PDR, 2023), the government has taken three key approaches towards donor transition: mobilizing domestic resources, increasing efficiency across programs and prioritizing primary health care (PHC) (World Health Organization, 2018).

### Domestic resource mobilization

**Figure 2.**
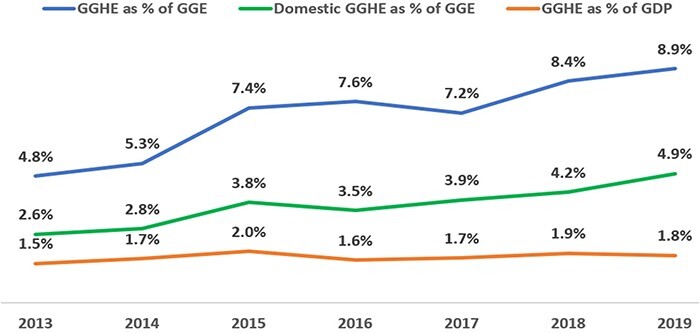
General government health expenditure as a share of government budget and GDP (%), 2013–2019

Note: GGHE, General Government Health Expenditure; GGE, General Government Expenditure; GDP, Gross Domestic Products. Source: (Ministry of Health of Lao PDR, 2021b).

Since 2013, there has been an increase of government funding for health (Figure 2). As shown in Figure 2, domestic government health expenditures as a share of the government expenditure increased from 2.6% in 2013 to 4.9% in 2019 (Ministry of Health of Lao PDR, 2021b). Moreover, the MOH has continued to advocate for greater investment in health from the government. This includes co-financing to sustain EHS in the context of donor transition and pro-health taxes in the Financing Strategy for the 9^th^ National Socio-Economic Development Plan 2021–2025 (The government of Lao PDR, 2023; 2021).

There are several models of co-financing in Lao PDR, including from the Vaccine Alliance (Gavi), Global Fund to Fight AIDS, Tuberculosis and Malaria (Global Fund), LuxDev and United Nations Population Fund. One model is the Lao-Luxembourg’s Joint Participatory Mechanism (JPM), which has annually multiplied government budgets for delivery of maternal and child health in three provinces since 2018. The multiplier formula, 20% MOH and 80% LuxDev, is applied based on well-developed budget-activity plans, timely budget disbursements by the government, and detailed indicators on quality-of-service delivery. This arrangement aims at facilitating the budget process for timely, effective and sufficient operational funding at the start of the fiscal year (Haegeman, 2019) and aligns the support with the planning and execution process (Heimann and Blaakman, 2022).

Tobacco tax has also been implemented with a decree on tobacco control fund stating that tobacco tax revenue will be used for priority health services and NHI in Lao PDR However, industry interference remains a challenge (Amul and Pang, 2018).

### Improving cross-programmatic efficiency

**Figure 3.**
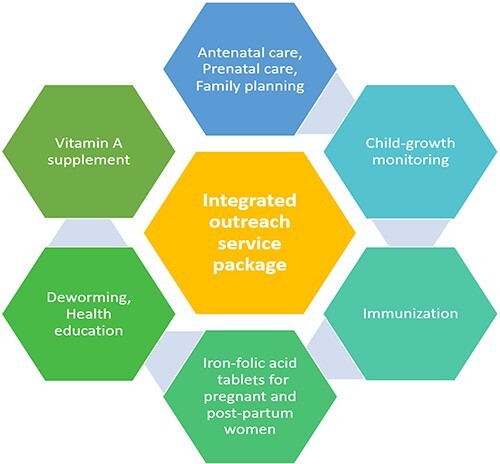
Integrated outreach package in Lao PDR

Source: (World Bank, 2017).

MOH initiated several models to improve efficiency across donor-funded programs by reducing fragmentation, duplication and misalignment. One such way has been to integrate joint outreach services of several maternal and child health, immunization and nutrition interventions as depicted in Figure 3 (World Bank, 2017). However, one assessment found that the lack of appropriate medical tools and equipment for an integrated outreach, along with misconceptions and various outreach incentives, were bottlenecks to this approach (World Bank, 2017).

Furthermore, between 2014 and 2016, the District Health Information Software2 (DHIS2) was rolled out nationwide and became the recognized health management information system platform by Ministerial Decree in 2017. The DHIS2 is a core platform that integrates the key health information systems to allow cross-program data sharing and use (World Health Organization, 2018). Almost all key public health programs, including separate disease surveillance systems, have been integrated into this platform as of the end of 2016.

Finally, the government has strived to improve the efficiency of funding management mechanisms by strengthening public financial management (PFM) and NHI. This has involved the development of a coherent policy between central and local governments, aligning provider incentives with PFM and the coordination of external funding. An additional mechanism to improve efficiency that is currently being explored is the expansion of NHI to cover vertically funded programs, Maternal and child health is already covered by NHI with a copayment exemption.

### Prioritization of PHC

The government is committed to strengthening PHC, a core foundation to achieve universal health coverage (UHC), through improving efficiency, resilience and sustainability of the health system (Ministry of Health of Lao PDR, 2019; 2020a; 2020b; 2021a; 2023). This commitment is aligned with the government’s decentralization policy (The government of Lao PDR, 2012). Additionally, in order to prioritize the evolving health needs of communities, MOH developed the Essential Health Service Package in 2018.

Lessons can be learned from the Health and Nutrition Services Access (HANSA) project, funded by the World Bank, Global Fund and Australia Department of Foreign Affairs and Trade from 2020 (World Bank, 2020; World Health Organization, 2022b). This employs a performance-based instrument and aims to improve performance at the health centre level as well as reduce fragmentation through joint financing towards health system strengthening (HSS) through PHC. It also aims at supporting preparations for transition from Global Fund to domestic financing by enhancing the existing system’s capacity to deliver TB/HIV and other services, specifically at the PHC level.

## Achievements/challenges

Donor transition has direct effects on the availability of financing human resources, medicines and service delivery in the health system (Kallenberg *et al.*, 2016; Teixeira *et al.*, 2017; Huffstetler *et al.*, 2022). Considering this, Lao PDR employed strategies that led to channelling external assistance towards HSS, such as aligning service delivery of vertical programs, merging health information systems and integrating maternal and child health into NHI. By prioritizing PHC, partners have shifted their support towards these efforts.

During transition, donors often require co-financing from the government to increase ownership and accountability and to facilitate long-term sustainability of previously vertically funded programs (World Health Organization, 2018). However, challenges are persistent, constraining progress toward a strong and sustainable health system. It was found that JPM, as mentioned above, raised concerns over the equitable resource allocation provided across provinces. The MOH needs to allocate earmarked co-financing budget for these selected provinces (crowding out the budget for other program activities), which puts pressure on the government to meet co-financing requirements during the economic crisis. Furthermore, with the economic crisis and the underfunded NHI, integrating services from vertical programs to NHI is no longer feasible (Ministry of Health of Lao PDR, 2022), unlike in examples from the Republic of Korea, the Philippines and Viet Nam (World Health Organization, 2018). Additionally, earmarking pro-health tax revenues is often considered to secure or increase public funding for health, but this only works in the short-term (Prakongsai *et al.*, 2008; World Health Organization, 2016; 2018; Cashin *et al.*, 2017).

Performance-based financing, pioneered in various countries (Lannes *et al.*, 2016; Renmans *et al.*, 2016; Ridde *et al.*, 2018), is conceptualized as ‘a tool for facilitating better, more inclusive, and more accessible health services’, which may have the potential to support system-wide reforms through increased motivation and financial management (Meessen *et al.*, 2011; Fritsche *et al.*, 2014; Paul *et al.*, 2018). However, because it can be implemented under donor pressure, there are concerns that it does not enable system reforms (Paul *et al.*, 2018). Attention is drawn to short-term results with huge opportunity costs of complicated management and verification mechanisms, which may actually weaken health systems (Valters and Whitty, 2017; Paul *et al.*, 2018). The implementation of HANSA was during the pandemic and is still at a nascent stage, thus it is difficult to extract conclusive evidence on the impact HANSA has had on PHC strengthening. Implementing performance-based financing will require careful design given the current economic situation and the constrained capacity of the current health system.

Translating HSS approaches into practice is challenging. Improving cross-programmatic efficiency is still conceptual and remains without concrete actions in many health centres. For instance, while integrated health information supports the efficient use of limited financial resources, the use of data remains limited. Additionally, donors still require specific financing and reporting systems, as seen with village health volunteers, and funds are transferred to health centres with insufficient prior capacity building efforts around planning, execution and monitoring.

Moreover, the economic crisis has hindered the government’s commitment to increase domestic resources, especially for PHC. Domestic government spending on PHC was 23.3% in 2019, and highly dependent on donor funding (26.7%) and out-of-pocket payment (50%), jeopardizing sustainability of EHS and medicines during the transition (Ministry of Health of Lao PDR, 2021b). However, limited domestic resources are still channelled towards big hospitals, which receive more attention due to their autonomy and private sector investments (Ministry of Health of Viet Nam, World Bank, 2011; Ravaghi *et al.*, 2018).

Critical to the success of decentralization is a strong system to share domestic and external financial information and funding flows across all levels of the system. Receiving financial information at subnational levels and getting involved in local planning, monitoring and evaluation remain a challenge. While the government has engaged with partners to align with government priorities and shift to a longer-term systems approach, much work remains to be done.

## Enablers/constraints

MOH plans to strengthen the health financing dialogue by establishing an inter-ministerial platform to discuss co-financing in order to enable sustained domestic funding for health.

Furthermore, lessons can be learned from previous efforts to strengthen the district health system, which could help strengthen PHC and sustain EHS. In the context of resource constraints, evidence-based planning is essential to efficiently deploy scarce resources to areas where they are most needed. In order to identify priority PHC facilities and repurpose critical functions, MOH plans to use all relevant geographically mapped data, including the demographic and socio-economic status of catchment areas, location of health facilities, the utilization of health services and resources available, such as staff. This can also lead to strategic staff planning and distribution at the subnational level.

Many donors (e.g. Japan International Cooperation Agency, n.d. and Korea Foundation for International Healthcare) have shifted their policies towards HSS (e.g. improving quality of health care and supporting NHI (Japan International Cooperation Agency, n.d.; Kim *et al.*, 2019)). Additionally, JPM and HANSA have used new HSS focused financing approaches (World Bank, 2020; Heimann and Blaakman, 2022); however, further evaluation is needed on their effectiveness, as previously mentioned. Various other models of external assistance for health (output-based and input-based financing) have been implemented (World Bank, 2020; Heimann and Blaakman, 2022), however, there needs to be sufficient support for this process of moving from input-based to output-based approaches in limited resource settings.

Government expenditure on social sectors accounted for 4.1% of GDP (1% in health) in 2021, which was well below the average compared to neighbouring countries and is not sufficient for the country’s needs (The government of Lao PDR, 2023). Suboptimal domestic funding is exacerbated by weak financial management capacity, inefficiency in resource allocations and delayed NHI reimbursement (Ministry of Health of Lao PDR, 2021a).

Like other countries, the current economic crisis in Lao PDR, contraction of health budgets and impending donor transition present a serious threat to sustaining EHS (Shaw and Varentsov, 2016; Huffstetler *et al.*, 2022; UNICEF Sri Lanka, 2023). In this context, donor transition plans proposed by donors will not work since they are based on the assumption that the country will grow wealthier and increase funding for the health sector. This situation suggests that GNI per capita is insufficient to evaluate a country’s readiness and capacity in the health system (Kallenberg *et al.*, 2016). The criteria need to consider government’s overall capacity and fiscal space for health, including the share of government expenditure on health or social sectors, tax revenue as a share of GDP, government debt level and maturity of the health system.

## Conclusions

Donor transitions triggered by economic growth can provide an opportunity for health system reforms. For securing the delivery of EHS as part of a well-performing, efficient health system, actions should be taken at the system rather than the program level. These actions include reducing fragmentation by way of pooling funds, allocating funds strategically and integrating overlapping services (Kutzin *et al.*, 2018).

The donor transition criteria need to consider more than just GNI per capita (Kallenberg *et al.*, 2016) and include government’s overall capacity and fiscal space for health, including the share of government expenditure on health or social sectors, tax revenue as a share of GDP, government debt level and how mature the health system is. This would allow for a more country-tailored approach that considers the local context and system readiness during the donor transition discourse. This paper contributes critical steps needed to prepare for transition: (1) advocating for the mobilization of critical resources; and (2) gaining efficiency through cross-programmatic alignment efforts and pooling donor funding around PHC. In the future, joint efforts by the government and partners will be needed to evaluate systematically what worked and what did not when it comes to effectiveness, equity, sustainability and efficiency. This will provide a critical basis for the government and partners to identify the optimal approaches for future transition plans and enhance coordination mechanisms with partners.

## Reflexivity Statement

The authors include three females and two males from three countries (Lao PDR, Germany and the Republic of Korea) and span across multiple levels of seniority. The first author specializes in health economics, the second author is a health system expert, the third author is a public health expert with more than 30 years of experience and the fourth and fifth authors are government officers in the Ministry of Health of Lao PDR with extensive experience in health policy and systems.
